# Nutrient availability and plant phenological stage influence the substrate microbiome in container-grown *Impatiens walleriana* ‘Xtreme Red’

**DOI:** 10.1186/s12870-024-04854-7

**Published:** 2024-03-06

**Authors:** Juan Quijia Pillajo, Laura J. Chapin, Cristian D. Quiroz-Moreno, James E. Altland, Michelle L. Jones

**Affiliations:** 1 Department of Horticulture and Crop Science, The Ohio State University, Wooster, OH 44691 USA; 2 Department of Horticulture and Crop Science, The Ohio State University, Columbus, OH 43210 USA; 3 Application Technology Research Unit, United States Department of Agriculture (USDA)-Agricultural Research Service, Wooster, OH 44691 USA

**Keywords:** 16S rRNA, Beta diversity, Fertilizer, Floriculture, Microbiota, Peat

## Abstract

**Background:**

The microbiome plays a fundamental role in plant health and performance. Soil serves as a reservoir of microbial diversity where plants attract microorganisms via root exudates. The soil has an important impact on the composition of the rhizosphere microbiome, but greenhouse ornamental plants are commonly grown in soilless substrates. While soil microbiomes have been extensively studied in traditional agriculture to improve plant performance, health, and sustainability, information about the microbiomes of soilless substrates is still limited. Thus, we conducted an experiment to explore the microbiome of a peat-based substrate used in container production of *Impatiens walleriana*, a popular greenhouse ornamental plant. We investigated the effects of plant phenological stage and fertilization level on the substrate microbiome.

**Results:**

Impatiens plants grown under low fertilization rates were smaller and produced more flowers than plants grown under optimum and high fertilization. The top five bacterial phyla present in the substrate were *Proteobacteria*, *Actinobacteria, Bacteriodota, Verrucomicrobiota,* and *Planctomycetota*. We found a total of 2,535 amplicon sequence variants (ASV) grouped into 299 genera. The substrate core microbiome was represented by only 1.8% (48) of the identified ASV. The microbiome community composition was influenced by plant phenological stage and fertilizer levels. Phenological stage exhibited a stronger influence on microbiome composition than fertilizer levels. Differential abundance analysis using DESeq2 identified more ASVs significantly affected (enriched or depleted) in the high fertilizer levels at flowering. As observed for community composition, the effect of plant phenological stage on microbial community function was stronger than fertilizer level. Phenological stage and fertilizer treatments did not affect alpha-diversity in the substrate.

**Conclusions:**

In container-grown ornamental plants, the substrate serves as the main microbial reservoir for the plant, and the plant and agricultural inputs (fertilization) modulate the microbial community structure and function of the substrate. The differences observed in substrate microbiome composition across plant phenological stage were explained by pH, total organic carbon (TOC) and fluoride, and across fertilizer levels by pH and phosphate (PO_4_). Our project provides an initial diversity profile of the bacteria occurring in soilless substrates, an underexplored source of microbial diversity.

**Supplementary Information:**

The online version contains supplementary material available at 10.1186/s12870-024-04854-7.

## Background

The floriculture industry relies on soilless substrates and chemical fertilizers to grow high-quality and marketable-sized plants at the right time of the year. Soilless substrates are mixes of organic (peat, coconut coir, or bark) and inorganic (perlite, sand, or vermiculite) materials designed to optimize plant growth and development [1]. Due to the limited nutrient holding capacity of soilless substrates, nutrients provided through fertilization (i.e., phosphate and sulfate) can be easily leached out of the container with irrigation [2, 3]. Intensive fertilization is required to compensate for nutrient losses from the container due to leaching, but this excessive nutrient application also increases production costs and contributes to the pollution of surface and groundwater sources. Recently, multiple approaches have been investigated for reducing fertilizer use and nutrient leaching, including revising and reducing recommended fertilization rates [4], identifying alternative nutrient sources [5], engineering alternative substrates [6], and utilizing plant-growth promoting rhizobacteria (PGPR) [7].

High quality ornamentals can be produced with a 60% reduction of the fertilizer recommended dose (150 ppm N) when plants are inoculated with PGPR [7]. *Petunia* × *hybrida* ‘Picobella Blue’ (petunia) inoculated with *Caballeronia zhejiangensis* strain C7B12 were larger and more floriferous than non-inoculated petunias receiving a two-fold higher fertilizer dose [8]. Recent research has shown that it is not only PGPR, but also their associated plant microbial communities that play an important role in modulating plant nutrient uptake, greenhouse gas emissions, and nutrient leaching [9, 10]. Moreover, microbial community transplant has been reported to alter flowering time [11], which is an important trait for greenhouse ornamental plants. Thus, capitalizing on the plant associated microbial communities has the potential to contribute to minimizing the use of nonrenewable inputs (i.e., chemical fertilizers) and reducing growing cycles in controlled environment production systems [8, 12].

The term microbiome refers to the microbial communities, including their genomic material, present in a specific ecosystem or host [13]. The soil microbiome is the most diverse on Earth and serves as the main source of microbial diversity in land-based ecosystems [14]. As plants grow, roots secret organic and inorganic compounds into the rhizosphere (the narrow area of soil surrounding the roots) to modify the chemical and biological properties of soil [15, 16]. Microbiome assembly at the rhizosphere is driven by the composition of root exudates, which is influenced by abiotic (i.e., nutrient deficiency) and biotic factors (i.e., pathogen attack) [17, 18]. The rhizosphere microbiome plays an important role in plant nutrition, development, and health [9, 18]. For instance, plant microbiome members participate in nitrogen fixation [19], nutrient solubilization (phosphorus, potassium and zinc) [20, 21], siderophore production [22], phytohormone production [23], pathogen biocontrol, [24] and induced systemic resistance (ISR) [25].

In contrast to the vast microbial diversity of soil [26], the microbial diversity of soilless substrate is greatly reduced [27]. Montagne et al. [27] reported that *Actinobacteria, Proteobacteria, Bacteroidetes* dominate the microbiome of bulk coco fiber, wood fiber, and peat. Similarly, Valles-Ramirez et al. [28] reported *Actinobacteria, Proteobacteria, Bacteroidetes, Chloroflexi,* and *Acidobacteria* as dominant bacterial phyla in leaf compost, pine bark, and peat. The fungal microbiome in soilless substrates is dominated by phyla *Ascomycota* and *Basidiomycota* [27, 28]. The substrate microbiome depends on the types of organic components (coir, wood fibers, peat, pine bark or leaf compost) and manufacturing processes (i.e., grinding technique and pH adjustment) [27, 28]. In bulk soil, the pH is the most important factor driving microbiome composition and diversity [29, 30]. In commercial substrate mixes, limestone is commonly added to adjust the pH to an optimal range for ornamental crop growth (5.4 to 6.8) [31]. Montagne et al. [27] also reported a change in microbiome composition when substrate pH was changed from 5.0 to 6.0. What or how other factors influence substrate microbiomes in controlled enviroment production systems is still unknown.

In container production, the substrate replaces the soil as the primary source of microorganisms for root microbiome assembly. Since substrate and soil microbiomes are different, they also drive the assembly of distinct rhizosphere microbiomes [32–34]. Bulk soilless substrates (peat and coconut fiber) are less diverse than soil [34]. Accordingly, plants grown in soilless substrates also assemble a rhizosphere microbiome less diverse than plants grown in soil [33]. However, plants grown in solid soilless substrates assemble rhizosphere microbiomes more diverse than plants grown in aeroponic and hydroponic systems [35]. Although soilless substrates have lower microbial diversity than soil, they do serve as a source of microorganisms for root microbiome assembly.

The root microbiome (rhizosphere and endosphere) is strongly influenced by soil type and host-genotype, with the soil serving as microbial source and the host-genotype-dependent selection of microorganisms [36]. In soil grown plants, the contribution of the soil microbiome to plant health and development is well-recognized [14]. In the greenhouse production of ornamental plants in containers, root development is restricted by the container size and shape. The primary sources of microorganisms in container culture are the substrate and water, but we still know very little about soilless substrate microbiomes and their influence on greenhouse ornamental plants. Developing microbial-based solutions to improve nutrient acquisition and plant visual quality requires a deep understanding of substrate and host-associated microbiomes and their responses to management practices such as fertilization [9, 37]. Therefore, the objective of this study was to describe the composition and diversity of bacterial communities associated with a peat-based substrate used for container culture of *Impatiens walleriana*, and to understand how these communities are influenced by fertilization rates.

## Materials and methods

### Plant material and greenhouse culture

*Impatiens walleriana* ‘Xtreme Red’ (impatiens) seeds (Syngenta Flowers, Gilroy, CA, USA) were sown in 288-size plug trays filled with a peat-based germination substrate (Pro-Mix PGX, Premier Tech Horticulture, Quakertown, PA, USA). Seedling trays were covered with a plastic dome to maintain humidity and germinated under fluorescent lights at room temperature (~ 21° C). Once the first true leaves expanded, the seedlings were fertilized with a rate of 25 mg per liter N (mg/L N) from a 15N-2.2P-12.5 K-2.9Ca-1.2 Mg water-soluble fertilizer (Jack’s Professional, JR Peters Inc., Allentown, PA, USA). Four weeks after sowing, the seedlings were transplanted into 15.2 cm round containers filled with a peat-based growing substrate (Pro-Mix BX, Premier Tech Horticulture), and watered with the corresponding fertilizer treatment solution. Plants were grown in a controlled environment greenhouse with a 14 h photoperiod and 21–24 °C day and 16–18 °C night temperatures.

Containers were organized in a randomized complete block design (RCBD) with six treatments and six blocks (*n* = 6). The treatments are described in Table 1 and are the combination of phenological stage and fertilizer level. Plants were harvested at two phenological stages: the beginning of the budding stage (four weeks after transplant), and when plants were fully flowering (eight weeks after transplant). The fertilizer treatments were prepared using 15N-2.2P-12.5 K-2.9Ca-1.2 Mg water-soluble fertilizer (Jack’s Professional, JR Peters Inc.,). Three fertilizer solutions were prepared that represented low (25 mg/L N), optimum (100 mg/L N) and high (200 mg/L N) fertilizer levels.

**Table 1:** Description of treatment combinations

Treatments	Phenological stage	Fertilizer level
1	Budding	Low (25 mg/L N)
2	Budding	Optimum (100 mg/L N)
3	Budding	High (200 mg/L N)
4	Flowering	Low (25 mg/L N)
5	Flowering	Optimum (100 mg/L N)
6	Flowering	High (200 mg/L N)

### Plant evaluation

Plant material and substrate samples were harvested at the budding (four weeks after transplant) and full flowering (eight weeks after transplant) stages. Open flowers, buds, and shoots were removed and placed in paper bags. Then, the root ball was removed from the container and vigorously shaken to separate the peat substrate from the roots. Samples of substrate were collected for microbiome and nutrient analyses (described below). The collected plant tissue was dried in a forced-air oven at 60 °C for at least 4 days and the dry weights were recorded.

### Substrate sampling

At harvest, most of the container area was occupied by the roots. We collected a subsample from all the substrate contained in the container after removing the roots, not only rhizosphere substrate. Thus, our sampling does not specifically distinguish between bulk and rhizosphere (substrate closely surrounding roots) substrate. Six substrate samples from each fertilizer treatment (low, optimum, and high) and at each phenological stage (budding and flowering) were collected in 2-ml microcentrifuge tubes and stored at -80 °C until DNA was extracted (*n* = 6 replicates/ treatment). In total, 36 samples were collected.

### Substrate nutrient analysis

After the substrate sample for sequencing was collected, a 200 ml substrate (aka media) sample was collected into plastic bags and stored for nutrient analyses. Samples were prepared using the saturated media extraction (SME) protocol [38]. Saturated media/ substrate extracts were filtered using a 0.45 µm nylon filter. Filtered samples were analyzed for pH, electrical conductivity (EC), total organic carbon (TOC), total dissolved nitrogen (TDN), and nutrient ions (NO, PO_4_, K, Ca, Mg, Cl, F, and SO_4_). Nutrient ions were measured using a Dionex ICS-6000 Ion Chromatograph (Thermo Fisher Scientific Inc., Waltham, MA, USA). TOC and TDN were measured using Shimadzu TOC-VCPH total organic carbon analyzer with TNM-1 TN measuring unit (Shimadzu Scientific Instruments, Columbia, MD, USA) [39].

### DNA extraction and sequencing

DNA was isolated from 250 mg of substrate samples using the DNeasy PowerSoil Kit (Qiagen, Germantown, MD, USA) according to the manufacturer instructions. DNA concentrations and quality were assessed using a spectrophotometer (NanoDrop ND-100, Thermo Fisher Scientific, Waltham, MA, USA). Extracted DNA was stored at -80 °C until it was sent for sequencing. 16S rRNA amplicon sequencing of the variable region V4 (V4_515F and V4_806) was conducted by Diversigen (New Brighton, MN, USA) [40].

### Statistical analysis and bioinformatics

Statistical analyses were conducted using R statistical software (R Foundation for Statistical Computing, Vienna, Austria). Normality and homoscedasticity of residuals were assessed before analysis of variance. One-way analysis of variance was conducted with shoot and flower dry weight according to the following model: $Y=\mu + treatment + block$. The significance level was set at α = 0.05.

Diversigen BenchMark is optimized for rhizosphere microbiome samples. Raw sequences were processed according to the DADA2 pipeline [41] and Diversigen BenchMark to generate the amplicon sequence variants (ASV). The Diversigen BenchMark provided the count and taxonomic tables for analysis.

All samples were rarefied to 31041 sequences per sample. Thus, sample LC8 was discarded due to insufficient sequence coverage. The core microbiome was identified using the rarefied dataset and the ‘microbiome’ package in R. An ASV was considered as core taxa if it was present in both collection times (100% persistence) and in all samples within a fertilizer treatment (100% prevalence) with at least 0.001% abundance. The sequences of core ASVs were used to construct a maximum likelihood phylogenetic tree with the ‘phangorn’ and ‘DECIPHER’ packages [42, 43]. The phylogenetic tree was visualized and annotated using the ‘treeio’, ‘ggtree’, and ‘ggtreeExtra’ packages [44–46].

The alpha-diversity represents the diversity of individual samples considering both richness and evenness. Richness refers to the number of different ASVs per sample, and evenness refers to the proportional distribution of ASVs in the sample. The estimation of Shannon and Simpson indices considers both richness and evenness. However, the Shannon index is more influenced by richness, whereas the Simpson index is more influenced by evenness [47, 48]. Richness, evenness, Shannon index, and Simpson index were calculated using the rarefied dataset and the ‘microbiome’ package in R. Statistical differences between groups were tested using the Kruskal–Wallis test (α = 0.05). If the Kruskal–Wallis test was significant (*p* ≤ 0.05), we performed pair-wise comparisons using the Wilcoxon test.

The relative abundance of bacterial phyla was presented as a stacked bar representation using the ‘microbiome’ package. Differences in community composition (beta-diversity) were visualized using a Bray & Curtis dissimilarity matrix and principal coordinate analysis using the ‘phyloseq’ and ‘vegan’ [49] packages. Differences in community composition were tested using the permutational analysis of variance (PERMANOVA) with the adonis function in the ‘vegan’ package [49].

The ‘DESeq2’ package was used for group-wise comparison using the model abundance ~ time * fertilization [50]. The non-rarefied dataset was used in the analysis. Only the five most abundant phyla were included in the analysis. ASVs present in less than 50% of the samples were removed. ASVs with a false discovery rate (FDR)-adjusted *p*-value of < 0.05 were selected as statistically significant.

Canonical correspondence analysis (CCA) was performed using the ‘vegan’ package. CCA was performed to model the effect of substrate nutrient levels on the substrate microbial community. Nutrient variables containing missing data were not included. The non-rarefied dataset was used in the analysis. Only the five most abundant phyla were included in the analysis. ASVs present in less than 50% of the samples were removed. The ASV count table was transformed to relative abundance. The most informative variables were chosen through a forward and reverse stepwise selection procedure based on the Akaike Information Criterion (AIC) using the ‘vegan’ package. PERMANOVA was performed to test the significance of our model and estimated marginal effect of each variable using the ‘vegan’ package. For the nutrients with a significant marginal effect, one-way analysis of variance was conducted according to the following model: $Y = \mu + block + treatment$. The significance level was set at α = 0.05.

Functional prediction was performed using the Functional Annotation of Prokaryotic Taxa (FAPROTAX) database [51] in the ‘microbiome’ package. FAPROTAX assigns ASVs to a functional group based on information available in the literature. Statistical differences between groups were tested using the Kruskal–Wallis test (α = 0.05).

## Results

### *Impatiens walleriana* ‘Xtreme Red’ performs well under limited fertilization

**Fig. 1:**
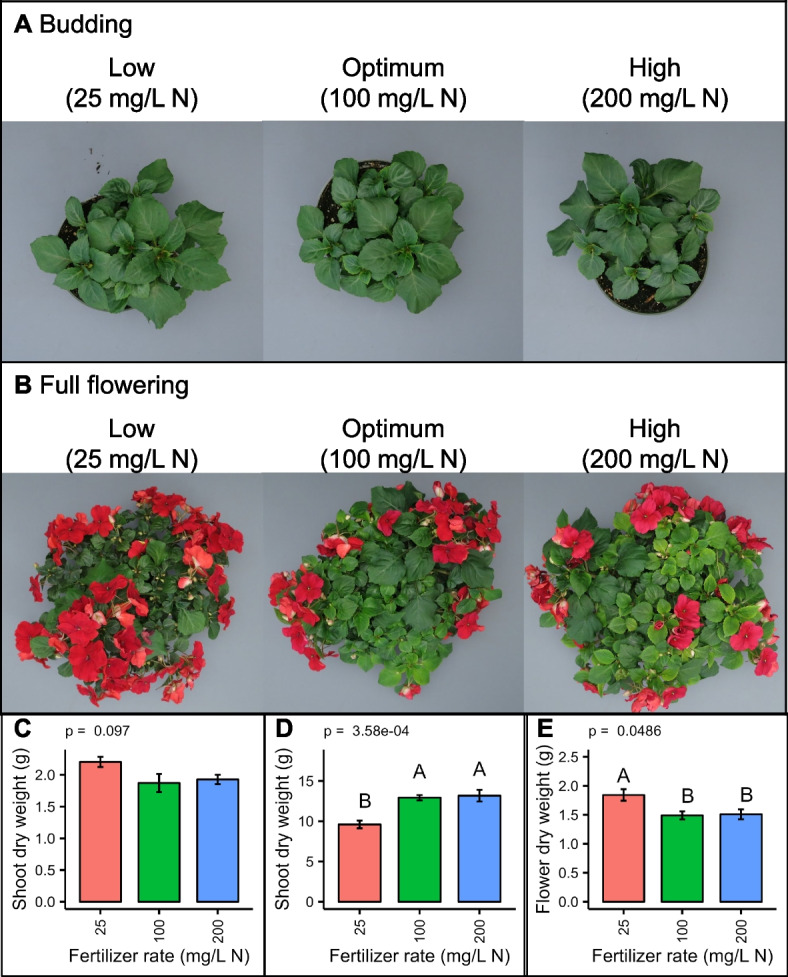
The effect of fertilization rate on the growth of *Impatiens walleriana* ‘Xtreme Red’. Plants fertilized with 15N-2.2P-12.5 K-2.9Ca-1.2 Mg water-soluble fertilizer at low (25 mg/L N), optimum (100 mg/L N), and high (200 mg/L N) levels. Pictures were taken at budding (**A**; four weeks after transplant) and full flowering (**B**; eight weeks after transplants). Shoot dry weight of plants harvested at budding (**C**). Shoot (**D**) and flower (**E**) dry weight of plants harvested at full flowering. Bars represent the mean ± standard error (*n* = 6). Bars sharing the same letter are not statistically different (*p* > 0.05) according to the Fisher LSD test

We cultivated *Impatiens walleriana* ‘Xtreme Red’ in peat-based soilless substrate under three fertilization levels: low (25 mg/L N), optimum (100 mg/L N), and high (200 mg/L N). At budding, the plants were not visually different (Fig. 1A). Accordingly, we did not find significant differences in shoot dry weight (*p* > 0.05) (Fig. 1C). At full flowering, we observed chlorosis on the optimum and high fertilization treatments (Fig. 1B). Plants under low fertilization did not show chlorosis but had lower shoot dry weight than optimum and high fertilization treatments (Fig. 1D). Interestingly, the low fertilization treatment showed higher flower dry weight than the other two treatments (Fig. 1E).

### The core microbiome of substrate harboring *Impatiens walleriana* ‘Xtreme Red’

**Fig. 2:**
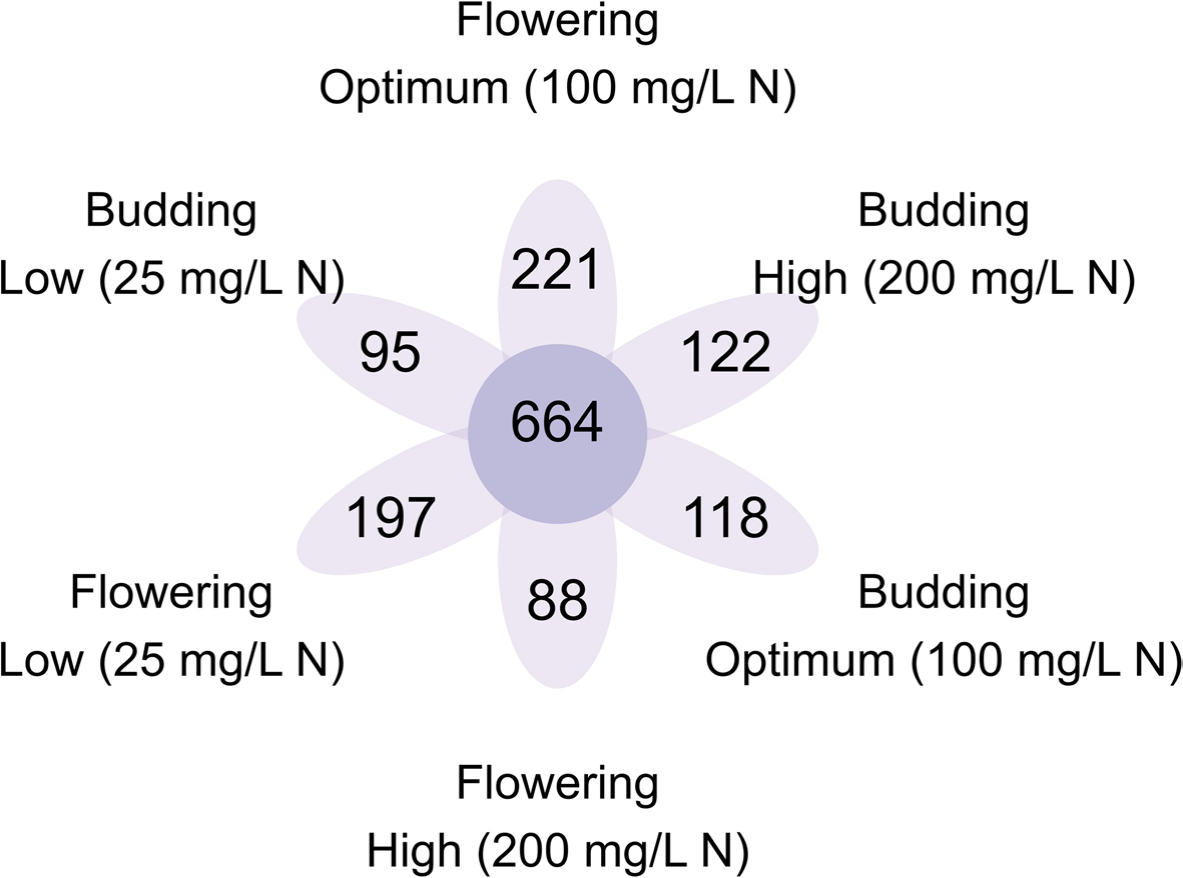
Venn diagram showing shared and unique amplicon sequence variants (ASV) identified in substrate samples. Samples were collected at budding (4 weeks after transplant) and full flowering (8 weeks after transplant), from container-grown *Impatiens walleriana* ‘Xtreme Red’ fertilized with 15N-2.2P-12.5 K-2.9Ca-1.2 Mg water-soluble fertilizer at 25, 100, or 200 mg/L of nitrogen (N)

For microbiome assessment, we sampled the substrate from container-grown impatiens ‘Xtreme Red’ plants that grew under the conditions described above. High-throughput sequencing of the 16S rRNA region from 35 samples produced 2,538,678 high-quality reads that grouped into 3,065 ASVs. After data cleaning (i.e., removing contaminant and spurious reads) we had 2,326,495 reads and 2,535 ASVs. There were 664 ASVs common among the six treatments. For samples collected at budding, we found 95, 118, and 122 unique ASVs for the low, optimum, and high fertilization treatments, respectively. For samples collected at full flowering, we found 197, 221, and 88 unique ASVs for the low, optimum, and high fertilization treatments, respectively (Fig. 2).

Since the number of ASVs per sample is strongly influenced by sequencing depth, rarefaction curves were constructed to evaluate the impact of sequencing depth on estimating species richness (Additional file 1). Samples were subsampled to 31,041 reads per sample to account for differences in sequencing depth and keep maximum data. The rarefied dataset contained 2,535 ASVs grouped into 24 phyla and 299 genera. Only about 10% of all reads were classified to the species level and about 75% to the genus level (Additional file 2).

**Fig. 3:**
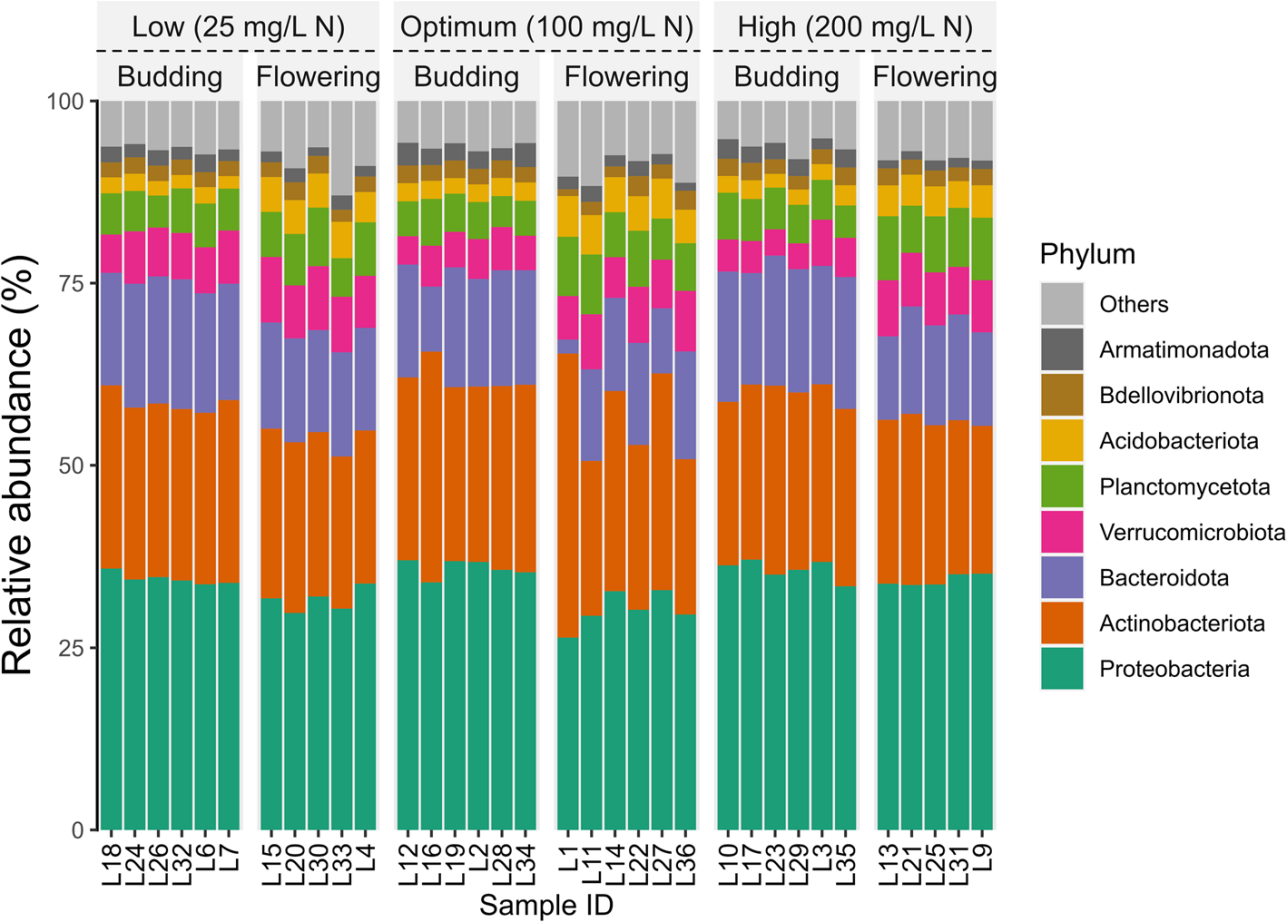
Relative abundance of bacterial phyla in substrate samples collected from container-grown *Impatiens walleriana* ‘Xtreme Red’. Plants were fertilized with 15N-2.2P-12.5 K-2.9Ca-1.2 Mg water-soluble fertilizer at low (25 mg/L N), optimum (100 mg/L N), and high (200 mg/L N) levels. Samples were collected at budding (four weeks after transplant) and full flowering (eight weeks after transplant). Only the top eight most abundant phyla are shown

**Fig. 4:**
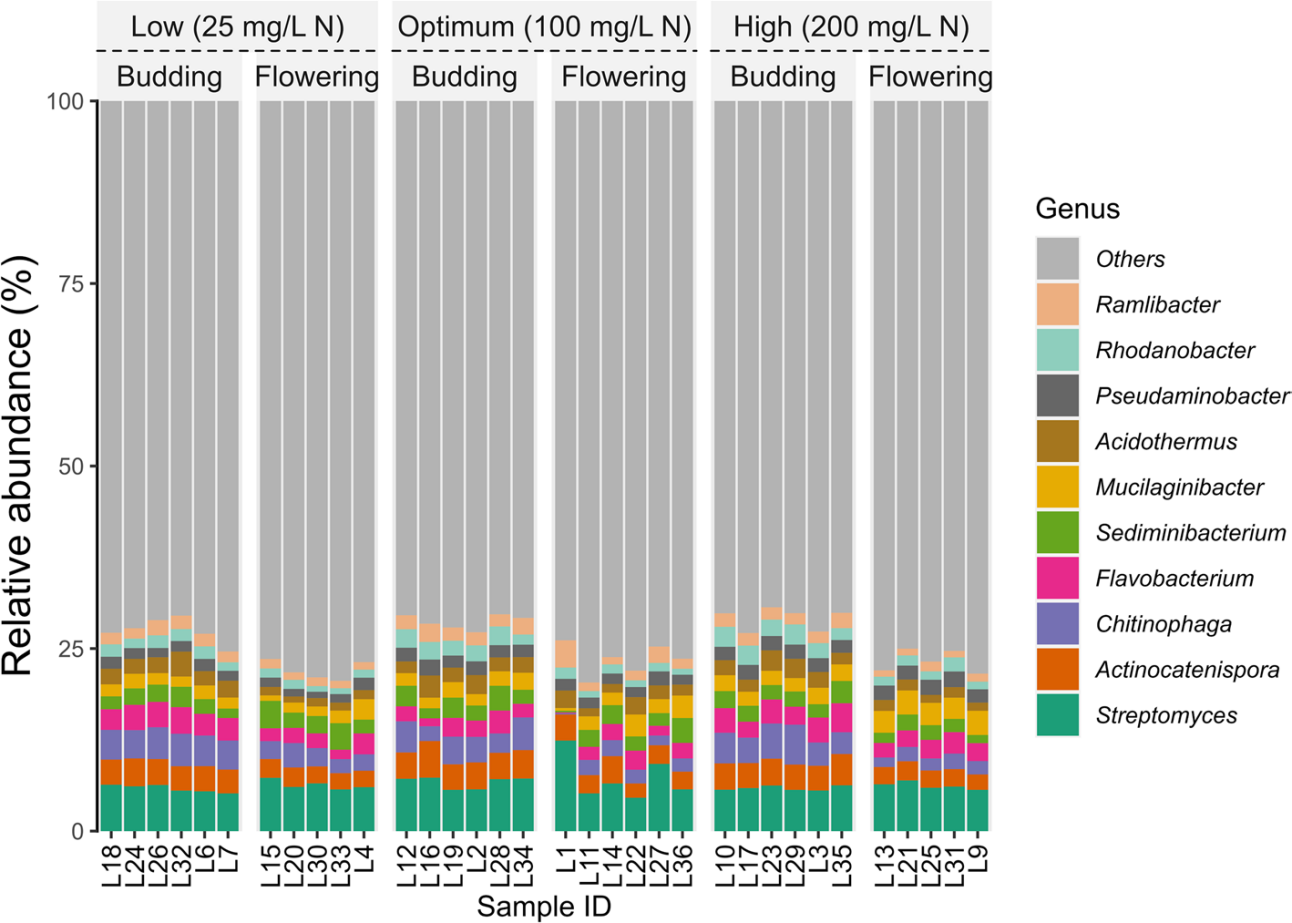
Relative abundance of bacterial genera in substrate samples collected from container-grown *Impatiens walleriana* ‘Xtreme Red’. Plants were fertilized with 15N-2.2P-12.5 K-2.9Ca-1.2 Mg water-soluble fertilizer at low (25 mg/L N), optimum (100 mg/L N), and high (200 mg/L N) levels. Samples were collected at budding (four weeks after transplant) and full flowering (eight weeks after transplant). Only the top 10 most abundant genera are shown

Community composition refers to the identity and relative abundance of different microbial taxa present in a particular ecosystem. In container-grown impatiens ‘Xtreme Red’, the top five taxa at the phyla level in the substrate microbial community were *Proteobacteria*, *Actinobacteria*, *Bacteriodota*, *Verrucomicrobiota*, and *Planctomycetota* (Fig. 3). At the genus levels, the top five taxa were *Streptomyces, Actinocatenispora, Chitinophaga, Flavobacterium,* and *Sediminibacterium* (Fig. 4).

**Fig. 5:**
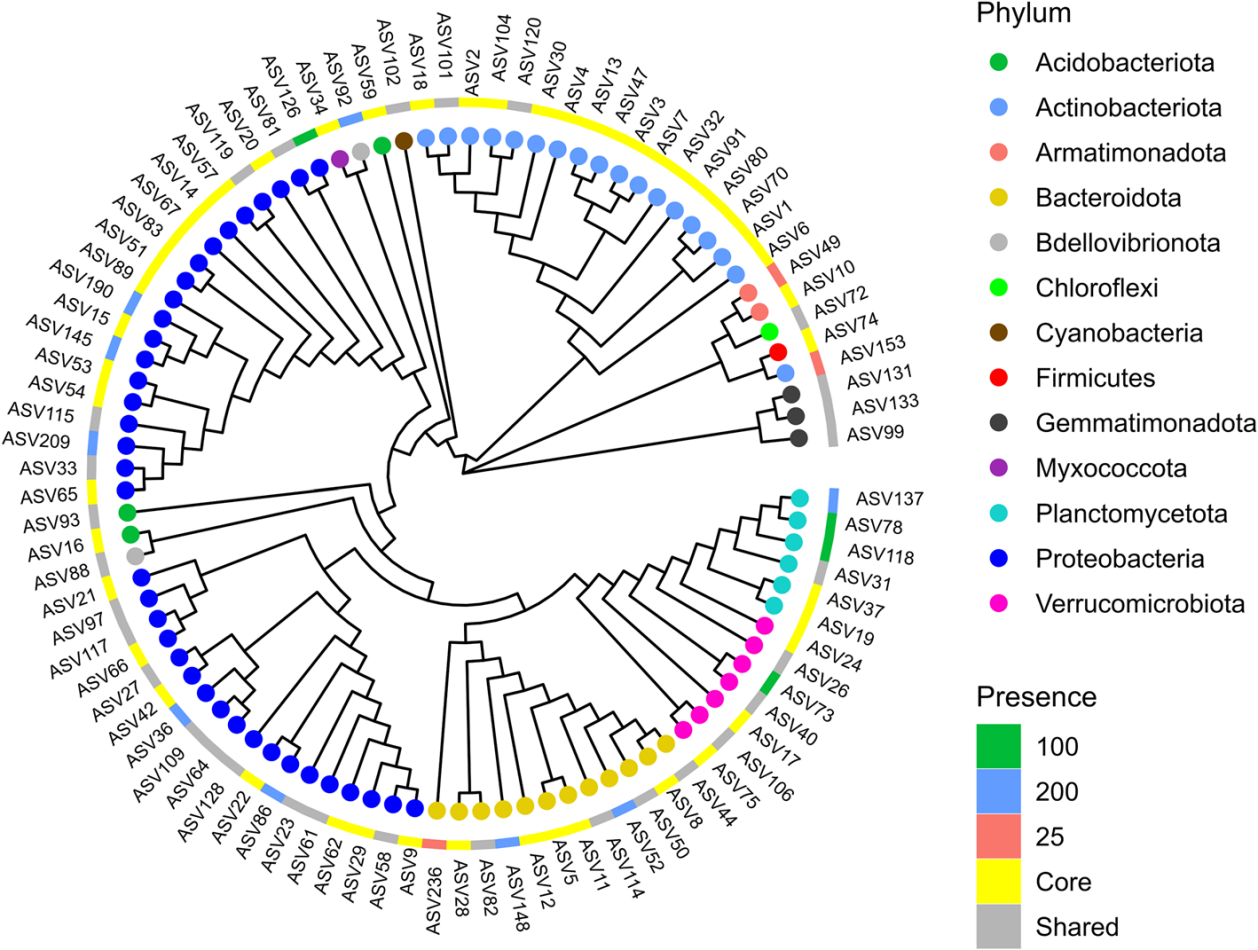
The core microbiome identified in substrate samples collected from container-grown *Impatiens walleriana* ‘Xtreme Red’. Plants were fertilized with 15N-2.2P-12.5 K-2.9Ca-1.2 Mg water-soluble fertilizer at low (25 mg/L N), optimum (100 mg/L N), and high (200 mg/L N) levels. Samples were collected at budding (four weeks after transplant) and full flowering (eight weeks after transplant). The core taxa were defined by 100% persistence (present in both collection time points), 100% prevalence (present in all samples of a treatment) and at least 0.001% abundance. Phylogenetic tree depicts taxa with 100% persistence, and tips are colored by phylum and annotated according to presence within the fertilizer treatments. Shared (color) refers to amplicon sequence variants present in at least two fertilizer treatments

The core microbiome refers to a set of microorganisms commonly found in a host or environment [52]. The core microbiome was defined at the ASV level by taxa with 100% persistence (present in both collection time points), 100% prevalence (present in all samples of a treatment) and at least 0.001% abundance. The substrate core microbiome included 48 core ASVs that belong to phyla *Proteobacteria* (18), *Actinobacteriota* (15), *Bacteroidota* (5), *Verrucomicrobiota* (3), *Planctomycetota* (2), *Acidobacteriota* (2), *Armatimonadota* (1), *Myxococcota* (1), and *Firmicutes* (1). Among the ASVs with 100% persistence, only three, four, and nine ASVs were unique for the low, optimum, and high fertilization treatments, respectively (Fig. 5).

### Plant phenological stage and fertilization levels influenced the substrate microbiome composition

**Fig. 6:**
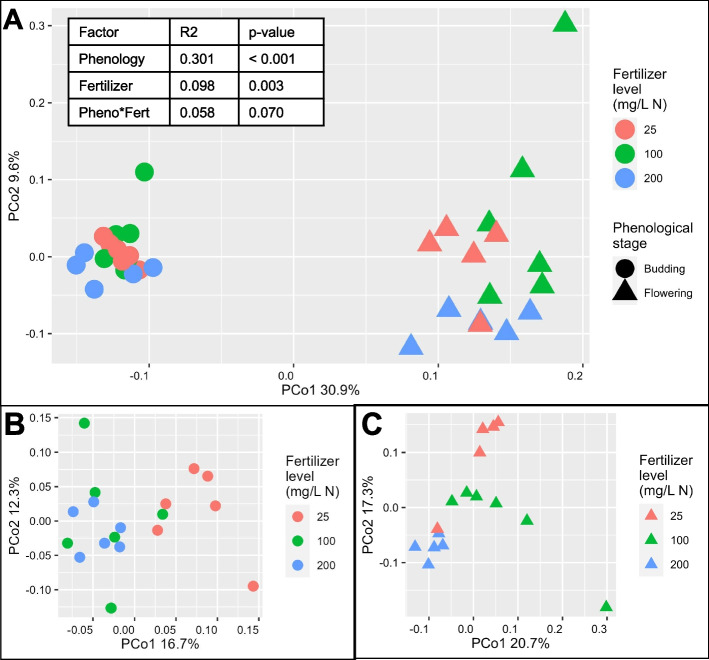
Beta diversity analysis of substrate samples collected from container-grown *Impatiens walleriana* ‘Xtreme Red’. Principal coordinate analysis was based on the Bray–Curtis dissimilarity matrix using the complete data set (**A**), samples collected at budding (**B**) and samples collected at full flowering (**C**). Plants were fertilized with 15N-2.2P-12.5 K-2.9Ca-1.2 Mg water-soluble fertilizer at low (25 mg/L N), optimum (100 mg/L N), and high (200 mg/L N) levels. Samples were collected at budding (four weeks after transplant) and full flowering (eight weeks after transplant). Phenological stage and fertilizer level effects were tested by conducting permutational analysis of variance (PERMANOVA) with the Bray–Curtis dissimilarity matrix

Developmental stages have been reported to influence the microbiome assembly of various crop and model plants [53, 54]. Accordingly, the substrate bacterial communities in samples collected from container-grown impatiens ‘Xtreme Red’ at budding and full flowering were also different. Principal coordinate analysis (PCoA) showed a clear separation between budding and flowering samples along the first principal coordinate (PCo1), which accounted for about 30.9% of the observed variance (Fig. 6A). Multivariate analysis of variance (PERMANOVA) also revealed a significant effect of phenological stage (*R*^2^ = 0.30111, df = 1, *p* < 0.0001) and fertilizer (*R*^2^ = 0.09805, df = 2, *p* = 0.00270), but the interaction effect was not significant (*R*^2^ = 0.05884, df = 2, *p* = 0.07349).

**Fig. 7:**
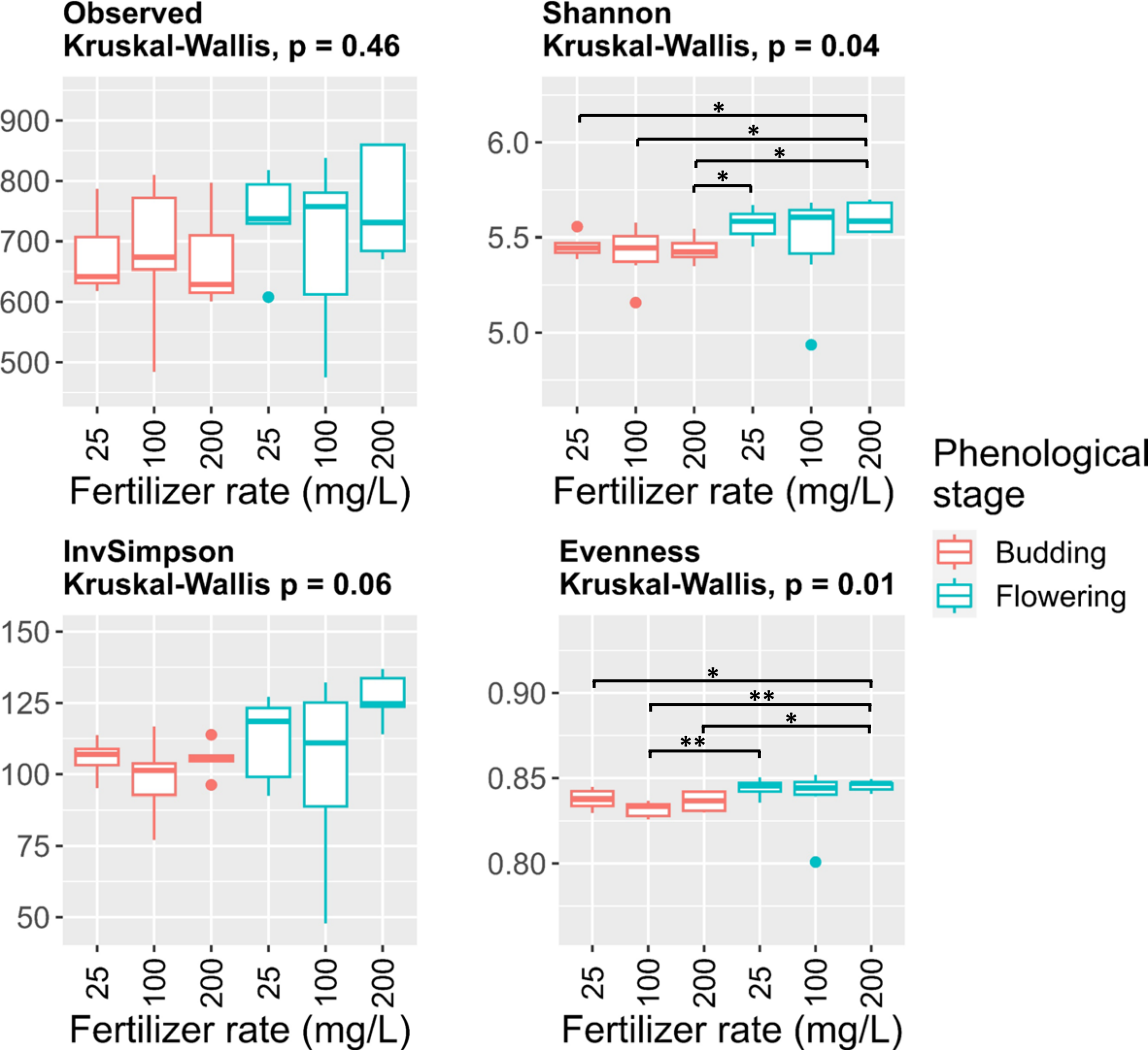
Richness, evenness, and alpha diversity of substrate samples collected from container-grown *Impatiens walleriana* ‘Xtreme Red’. Plants were fertilized with 15N-2.2P-12.5 K-2.9Ca-1.2 Mg water-soluble fertilizer at low (25 mg/L N), optimum (100 mg/L N), and high (200 mg/L N) levels. Samples were collected at budding (four weeks after transplant) and full flowering (eight weeks after transplant). For each function, differences between treatments were tested using the Kruskal–Wallis test. If the Kruskal–Wallis test was significant (*p* ≤ 0.05), we performed pair-wise comparisons using the Wilcoxon test (* 0.01 < *p* ≤ 0.05, ** 0.001 < *p* ≤ 0.01, *** *p* ≤ 0.001)

To look at the effects of nutrient availability on microbiome structure, we conducted individual PCoA analyses for each phenological stage. At budding, low fertilization samples clustered and separated from high fertilization. The separation was mainly along the first principal coordinate (PCo1), which accounted for about 16.7% of the observed variance (Fig. 6B). At full flowering, we better observed the shift of the substrate microbiome along the fertilizer gradient (Fig. 6C). Finally, we did not observe a clear significant effect of phenological stage or fertilization on richness, evenness, or alpha-diversity indices (Fig. 7).

**Fig. 8:**
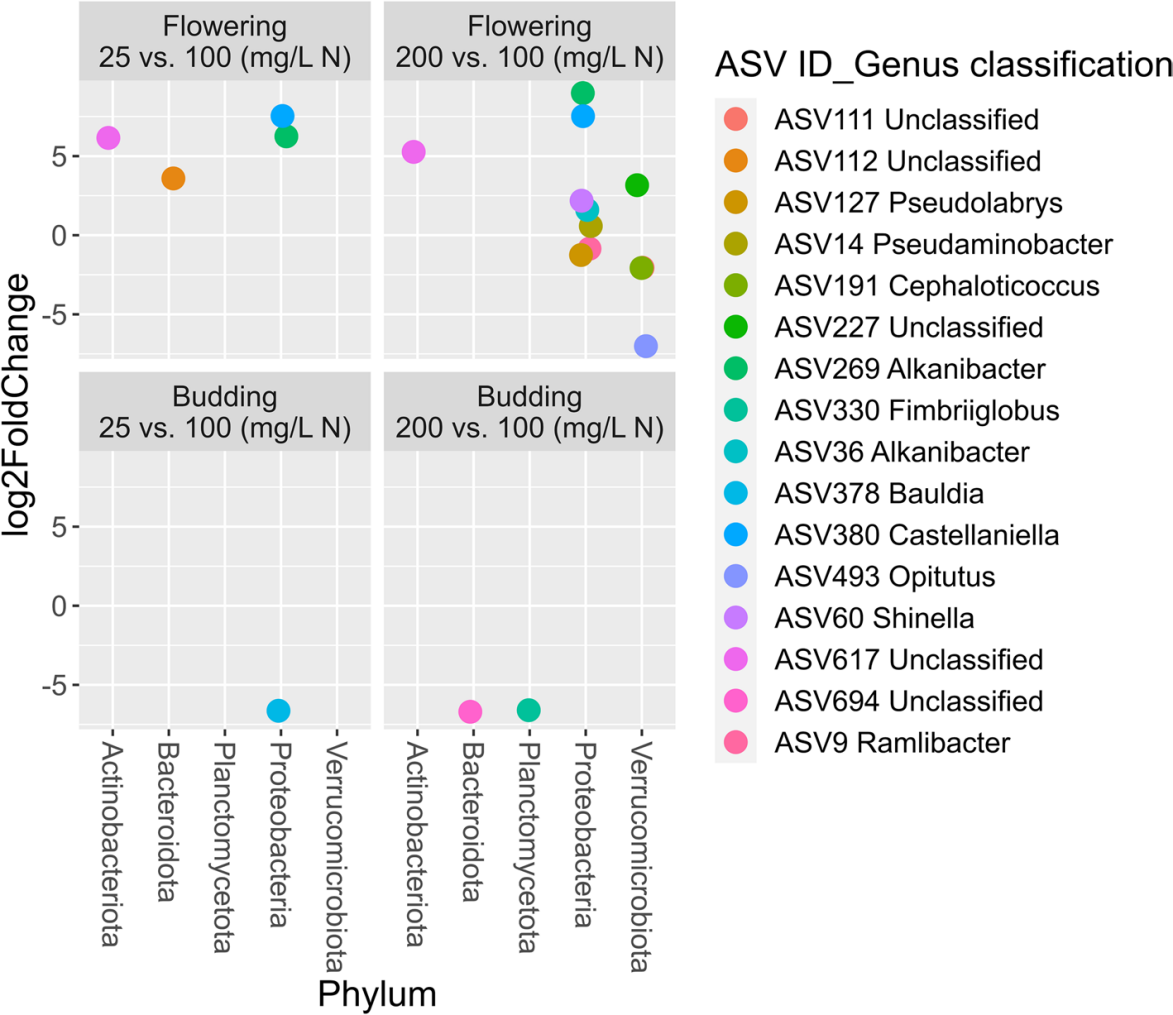
Differential abundance analysis (DESeq2) between substrate samples collected from container-grown *Impatiens walleriana* ‘Xtreme Red’. Plants were fertilized with 15N-2.2P-12.5 K-2.9Ca-1.2 Mg water-soluble fertilizer at low (25 mg/L N), optimum (100 mg/L N), and high (200 mg/L N) levels. Samples were collected at budding (four weeks after transplant) and full flowering (eight weeks after transplant). The DESeq2 analysis indicates the fold-change of abundance due to fertilizer treatments in comparison to the 100 mg/L N level at each collection timepoint. DESeq2 uses the Benjamini-Hochberg (BH) adjustment to correct for multiple testing, and only amplicon sequence variants (ASV) with an BH-adjusted *p* ≤ 0.05 are shown

As we were interested in the effect of fertilization on the substrate microbiome, we analyzed fertilization-dependent changes in the abundance of microbes in the rhizosphere. Differential abundance analysis using DESeq2 was conducted with taxa present in more than 50% of the samples with more than five reads. We used budding as reference level for the factor phenological stage and optimum as the reference level for the factor fertilizer level. We then identified microbial taxa significantly enriched in the low and high fertilizer levels at budding and full flowering (Fig. 8). At budding, we identified three ASVs. ASV378 was depleted at low fertilization, while ASV694 and ASV330 were depleted at high fertilization.

At flowering, ASV617, ASV380, and ASV269 were enriched in both low and high fertilization. ASV112 was enriched only at low fertilization. We found the higher number of differentially abundant taxa at high fertilization, where four ASVs were enriched (ASV14, ASV36, ASV60, ASV227) and five were depleted (ASV9, ASV111, ASV127, ASV191 and ASV493). None of the identified taxa were classified to the species level, and five were not even classified to the genus level.

**Fig. 9:**
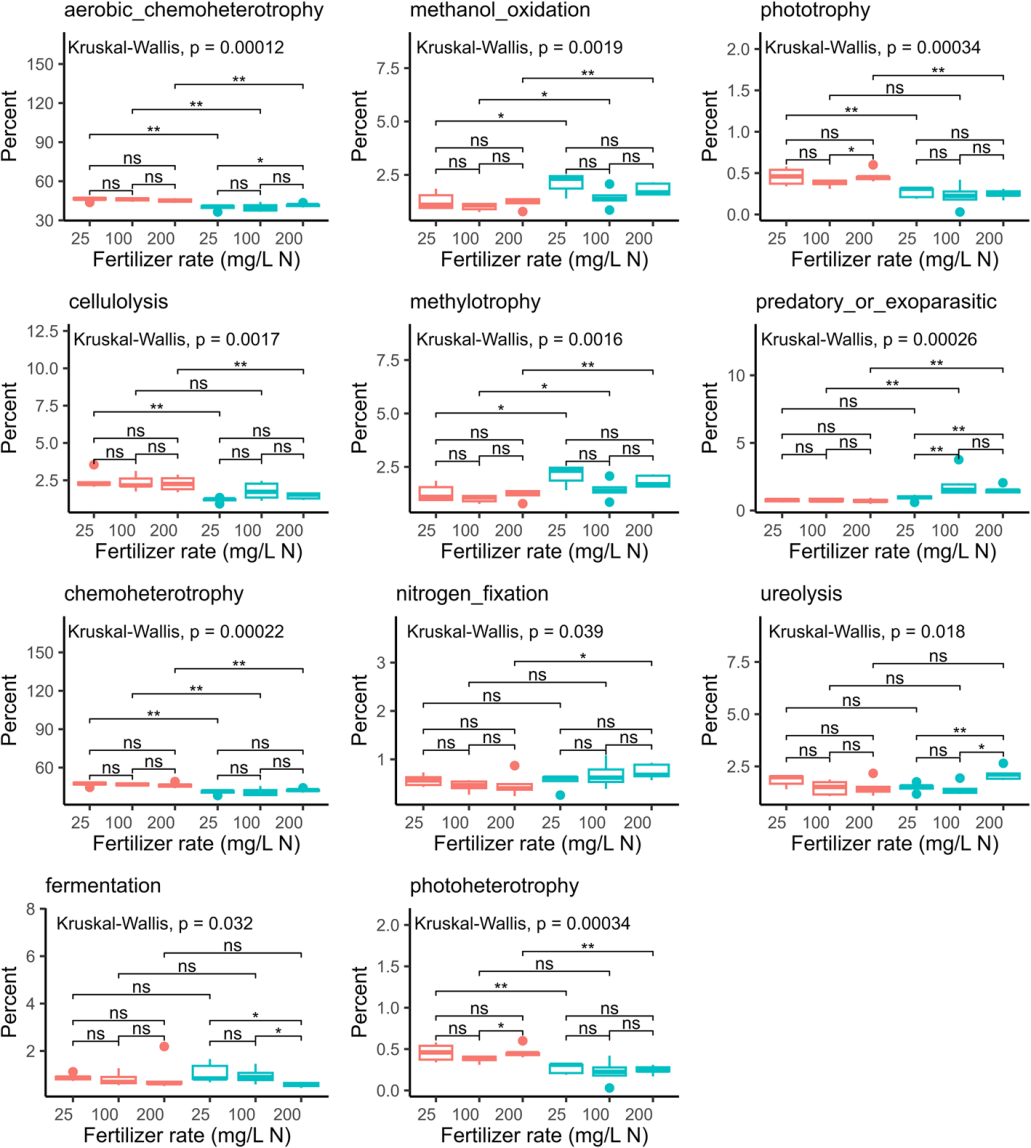
Relative abundance of bacterial functions identified in the substrate samples using the FAPROTAX database. The substrate samples were collected from container-grown *Impatiens walleriana* ‘Xtreme Red’ fertilized with 15N-2.2P-12.5 K-2.9Ca-1.2 Mg water-soluble fertilizer at 25, 100, and 200 mg/L of nitrogen (N). Samples were collected at budding (4 weeks after transplant) and full flowering (8 weeks after transplant). Only the top 11 most abundant functions are presented. For each function, differences between treatments were tested using the Kruskal–Wallis test. If the Kruskal–Wallis test was significant (*p* ≤ 0.05), we performed pair-wise comparisons using the Wilcoxon test (* 0.01 < *p* ≤ 0.05, ** 0.001 < *p* ≤ 0.01, *** *p* ≤ 0.001)

Nutrient limitation can influence soil microbial community function and drive the assembly of microbiomes that help maintain plant growth [55]. To look at the effects of fertilizer levels on community function, the ASVs were assigned to functional categories using the FAPROTAX database [51]. We selected the 15 most abundant functional groups for analysis. The top two functions identified were chemoheterotrophy and aerobic chemoheterotrophy. The Kruskal–Wallis test showed significant differences in 11 functions (Fig. 9). Pair-wise comparison revealed that differences in each function were mainly between the two phenological stages. We observed an effect of fertilization levels mostly at full flowering on aerobic-chemoheterotrophy, fermentation, predatory-or-exoparasitic, and ureolysis. At high fertilization levels, ureolysis was higher and fermentation was lower than at low fertilization. The proportion of reads classified as predatory or exoparasitic was lower in the low fertilization than in the optimum and high fertilization.

**Fig. 10:**
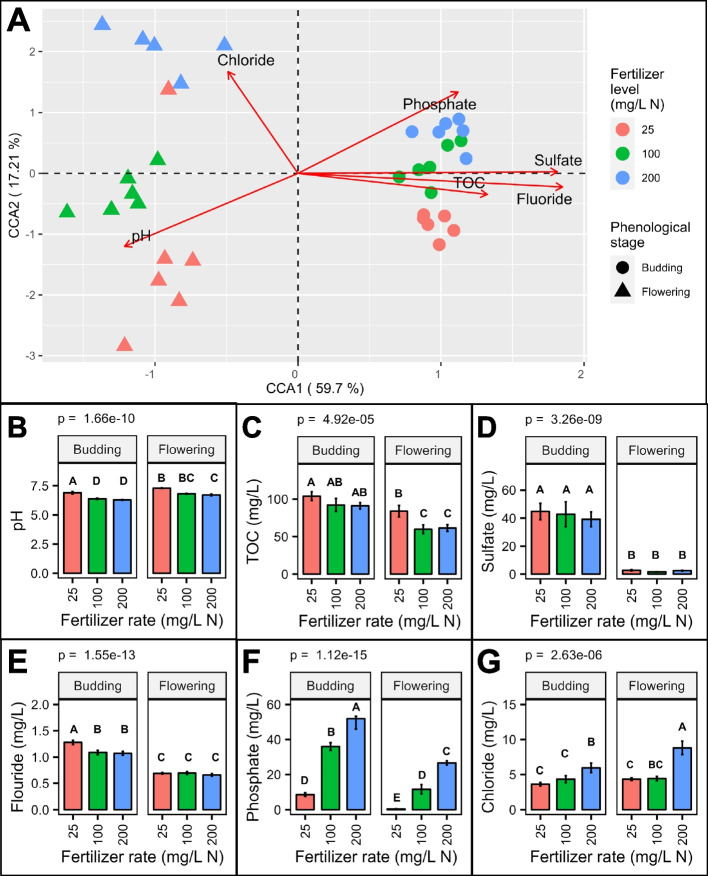
Association of environmental variables and bacterial community composition of the substrate. Canonical correspondence analysis (CCA) (**A**). The pH (**B**), total organic carbon (TOC) (**C**), sulfate (**D**), fluoride (**E**), phosphate (**F**), and chloride (**G**) concentration of the substrate. Substrate samples were collected from container-grown *Impatiens walleriana* ‘Xtreme Red’. Plants were fertilized with 15N-2.2P-12.5 K-2.9Ca-1.2 Mg water-soluble fertilizer at 25, 100, and 200 mg/L of nitrogen (N). Samples were collected at budding (4 weeks after transplant) and full flowering (8 weeks after transplant). Bars represent the mean ± standard error (*n* = 6). Bars sharing the same letter are not statistically different (*p* > 0.05) according to the Fisher least significant difference test

During sample collection, we also collected substrate samples from each container to characterize chemical properties (Additional file 3). Canonical correlation analysis was conducted to understand the relationship between substrate microbial communities and the chemical properties of the media (considered as explanatory variables). Explanatory variable selection was based on the variance inflation factor (VIF) and a stepwise selection procedure. VIF measures to what extent each variable in a data set is intercorrelated with other variables (collinearity). Five variables showed low collinearity (pH = 6.2, total organic carbon (TOC) = 3.3, fluoride (F) = 4.5, chloride (Cl) = 1.5 and sulfate (SO_4_) = 6.9). The stepwise selection procedure rendered a significant model with three environmental constraints [chloride (Cl), phosphate (PO_4_), and fluoride (F)]. Thus, our final model included the six explanatory variables (pH + TOC + F + Cl + SO_4_ + PO_4_), it was highly significant (*P* = 0.001), and it produced two significant canonical correspondent axes (CCA1: *p* = 0.0001 and CCA2: *p* = 0.015). The two first canonical axes explained 76.9% of the constrained variation in the substrate bacterial communities. CCA1 clearly distinguished the samples collected at budding from the ones collected at full flowering. The pH, TOC, and fluoride were significantly associated to the observed clustering along CC1 (Fig. 10A). Moreover, although sulfate was not significant, it was positively correlated with TOC and fluoride. Accordingly, there was a consistent difference in pH, TOC, sulfate, and fluoride concentration between samples collected at budding and flowering, but not between fertilizer treatments compared at each stage (Fig. 10B, C, D, E). Clustering according to the fertilizer levels was observed along CCA2. Cl (*p* = 0.016) was significantly associated to CCA2 (Fig. 10A). The pH was negatively correlated to phosphate, and they both appeared to partially contribute to clustering along CC2. Accordingly, we observed a significant phosphate gradient at both phenological stages (Fig. 10F). In addition, at each stage the lowest phosphate values were accompanied by the highest pH values (Fig. 10B), and chloride concentration increased at higher fertilizer levels (Fig. 10G).

## Discussion

Greenhouse ornamental plants are primarily grown in soilless substrates, and peatmoss is the most common component in greenhouse substrates [56]. Although various research efforts have focused on improving chemical [57–59] and physical [6, 60] properties of soilless substrates to enhance plant growth and product quality, less research has focused on the microbiological properties. In soil-based agriculture systems the soil serves as the primary source of microorganisms for plant microbiome assembly, and those microorganisms significantly contribute to plant health and development [15]. In substrate-based controlled environment agriculture, soil is replaced with commercially available soilless-substrates and this substrate serves as the source of microorganisms for the plant microbiome. However, the substrate microbiomes and their potential effects on the plant have not been fully explored. Due to the relevance of microorganisms in plant nutrient uptake and nutrient cycling [10], there is an increasing interest in studying the microbiomes of soilless substrates and their potential contributions to reducing the reliance on synthetic fertilizers. Thus, to gain insight into the microbiome of soilless substrates, we examined the microbiome of a peat-based substrate (80% peat and 20% perlite v/v) used to grow *Impatiens walleriana* ‘Xtreme Red’, a popular greenhouse ornamental plant. The substrate bacterial microbiome was assessed using 16S rRNA amplicon sequencing. This approach allows the identification of culturable and unculturable microorganisms and has been widely used to assess earth’s microbial diversity [61, 62].

*Proteobacteria*, *Actinobacteria*, and *Bacteriodota* are dominating groups in bulk peat, wood fiber, compost, pine bark, and coconut coir [27, 28]. Accordingly, those phyla also dominated the microbiome of a peat-based substrate used in container culture of impatiens ‘Xtreme Red’. The dominant phyla in tropical and boreal peatlands are *Proteobacteria* and *Acidobacteria* [63]. *Proteobacteria* is fast-growing and its abundance is positively correlated to carbon availability [64]. *Acidobacteria* are common inhabitants of acidic environments. Peatlands are ecosystems with high carbon content and acid pH (3.9 to 4.5), thus they provide conducive conditions for development of *Proteobacteria* and *Acidobacteria* [65]. Interestingly, we observed that *Acidobacteria* ranked sixth in our study. An important distinction is that the pH of commercial substrates is commonly higher than the pH of peatlands. Greenhouse substrates are commonly amended with limestone to reach the pH required (5.4 to 6.8) for the production of ornamental plant species [31]. Montagne et al. [27] reported that adjusting pH from 6.0 to 7.0 changed the microbiome composition and diversity of sphagnum peat. However, pH was adjusted by adding a phosphate solution (0.1 M K_2_HPO_4_, 0.01 M KH_2_PO_4_), thus it is unclear if the observed changes in the microbiome were only due to the pH shift or to the additional phosphorus and potassium added. Although the dominant phyla of our peat-based substrate was similar to the ones reported in other soilless substrates [27], we observed a more distinct substrate microbiome composition at the genus level which was dominated by *Streptomyces*, *Actinocatenispora*, *Chitinophaga,* and *Flavobacterium*. In contrast, Anzalone et al. [34] reported that the microbiome of coconut fiber was dominated by *Pseudonocardiaceae*, *Rhodococcus*, *Ochobactrum*, and *Bacillus,* and the microbiome of blonde sod peat was dominated by *Pedosphaeraceae*, *Desulfovibrio, and Flavobacterium*. Therefore, variability in microbiome composition can be found across commercial soilless substrates, and this variability is shown to be driven by factors such as the organic ingredients and manufacturing process [27]. Substrate-based soilless culture drives the assembly of distinct root microbiomes [34, 66], yet there is a gap in our knowledge about their potential functions. In substrate-based container production, the substrate serves as the main source of microorganisms for the root microbiome. However, while the bacterial richness of soil was estimated to reach up to 52 000 species per gram of soil [26], the reported bacterial richness of soilless substrates (peat, bark and coconut fiber) ranges from 38 to 124 species [27], and the bacterial richness of our peat-based substrate ranged from 500 to 800 species. Thus, root microbiomes of plants grown in soilless substrates can be limited to the available microbial diversity in the substrate.

Plants can modulate the soil/substrate microbiome. Underground, plants interact with soil microorganisms (or substrate microorganisms in our system) through the exudates released by the root into the rhizosphere [15]. The quality and quantity of the root exudates changes along the plant life cycle [67] and in response to biotic [68] and abiotic stress [69]. These changes in root exudation profiles in turn influence the diversity and composition of the soil microbiome. We did not observe changes in the substrate alpha-diversity associated with plant phenological stage or fertilizer level. Accordingly, no significant effects were reported in the alpha diversity of bulk substrate (80% white peat and 20% coconut fiber v/v) used to grown *Solanum melongena* ‘Jaylo’ [66]. Similarly, plant phenological stage did not affect soil alpha diversity [67]. On the other side, Grunert et al. [70] reported that application of struvite or organic fertilizer slightly changes alpha diversity when analyzing rhizosphere substrate. Our observations could be impacted by our sampling approach, as it consisted of a subsample of all media contained in the container after removing the roots, not only rhizosphere substrate.

Although we did not observe an effect of phenotypic stage or fertilization on alpha diversity, they both modulated microbial community composition. Phenological stage showed a strong effect on the bacterial community composition of the substrate (Fig. 6). The microbiome of substrate harboring budding impatiens was distinct from the microbiome of substrate harboring flowering impatiens, and substrate TOC, sulfate, and fluoride were significantly associated with the observed differences in microbiome composition. The phenology effect has also been reported in cotton, where the rhizosphere soil microbiomes were different at seedling, budding, and flowering stages in two cotton cultivars (*Gossypium hirsutum* L. ‘TM-1’ and *G. barbadense* L. ‘Hai 7124’) [71]. But more importantly, Panke-Buisse et al. [11] reported that flowering can be accelerated in late-flowering arabidopsis plants through soil microbiome transplant. Thus, microbiome harnessing opens a possibility to modulate plant phenology that could help greenhouse growers meet seasonal demands.

Growing high-quality ornamentals to meet seasonal demands requires continuous fertilization. Continuous application of synthetic fertilizer changes the microbial community composition and function of rhizosphere soil [72]. Accordingly, we also observed an effect of synthetic fertilization in the substrate microbiome. The substrate microbial community composition and function (aerobic-chemoheterotrophy, fermentation, predatory-or-exoparasitic, and ureolysis) changed in response to the amount of fertilizer applied through the watering solution. The effect of fertilization levels on microbial community composition were more apparent at flowering (eight weeks under continuous liquid fertilization), and chloride and pH were significantly associated with the distinct substrate microbiome compositions. The soil pH affects nutrient bio-availability [16], and is the most important factor driving soil microbiome composition and diversity [29, 30]. Plants can modify root exudate composition as a mechanism to cope with nutrient scarcity. Some of the exuded compounds can directly influence nutrient availability (i.e., coumarins) [73], and others serve as signaling molecules to drive the assembly of soil microbiomes, whose members can also contribute to increased nutrient availability (i.e., nitrogen fixers, phosphorus solubilizers, siderophore producers) [9, 74]. Thus, in the rhizosphere zone, microbiome composition and function are driven by the soil nutritional content and the plant response to nutrient availability in the soil [75]. Moreover, the microbiome effect on plant phenotype can shift from positive to negative by changes in the lifestyle of certain bacterial groups in response to nutrient availability. For instance, Finkel et al. [75] showed that a 185-member bacterial synthetic community (SynCom) promotes growth in arabidopsis under normal phosphorus nutrition, but not under phosphorus starvation. However, the growth promotion effect of the SymCom was recovered by removing *Burkholderia* members from the Sym-Com. The above observations highlight the potential value of microbiome engineering as a tool to reduce synthetic fertilization. Microbiome engineering for substrate-based controlled environment agriculture requires deeper study of the microbial ecology of substrates microbiomes, including their member identities, lifestyles, and functions.

Although substrate microbiomes could change in response to phenological stages and fertilizer levels, the species that were present in the substrate despite the applied treatments can be considered as the core microbiome of our peat-based media. The core microbiome refers to a set of microorganisms consistently found in a host or environment [52]. Due to their potential contribution to plant development and health, the rhizosphere core microbiome has been determined for various plant hosts like arabidopsis, rice, and corn [76, 77]. In substrate harboring impatiens plants, we found a core microbiome of 48 ASVs that were present in all samples from all treatments with an abundance of at least 0.001%. Other core microbiomes have been reported to contain seven (maize) [78], 97 (arabidopsis) [76], 88 (rice) [77] and 48 species (common bean) [79]. Moreover, *Proteobacteria* and *Actinobacteria* are the dominating phyla in the core microbiome of all the species mentioned above. Similarly, the majority of species (79%) in the core microbiome of substrate harboring impatiens belong to phyla *Proteobacteria*, *Actinobacteria*, and *Bacteriodota*.

To define which taxa at the ASV-level were influenced by fertilization levels we conducted a differential abundance analysis using DESeq2. We compared the low (25 mg/L N) and high (200 mg/L N) fertilization levels against the optimum level (100 mg/L N) at both collection timepoints (budding and flowering). At budding, no taxa were enriched by the fertilization treatments, but ASV378 was depleted by the low fertilization, and ASV330 and ASV694 were depleted by the high fertilization. ASV378 belongs to genus *Bauldia,* which is associated with nitrogen-fixation [80]. ASV330 belongs to genus *Fimbriiglobus,* which is reported as chitinolytic and cellulolytic bacteria [81, 82]. ASV694 belongs to family *Chitinophagaceae,* whose members are reported to produce chitinases and exhibit antifungal properties [83, 84].

At flowering, we found 16 ASVs significantly affected by the fertilization levels. ASV112, ASV269, ASV380, and ASV617 were enriched by low fertilization. ASV112 belongs to the candidate family env.OPS 17, phylum *Bacteroidota*, a taxonomic family present in ground water, but with few cultured isolates [85]. Interestingly, ASV269, ASV380, and ASV617 were enriched by both low and high fertilization. ASV269 and ASV380 belong to the genus *Alkanibacter* and *Castellaniella*, respectively. Species from genus *Castellaniella* are described as denitrifiers [86, 87], and *Alkanibacter* bacteria is able to degrade hexane [88]. ASV617 belongs to order *Gaiellales*, an order predominant in extreme environments like saline–alkali agricultural soil and marine ecosystems [89, 90]. ASV269 and ASV380 are *Proteobacteria*, and ASV617 is *Actinobacteria*, both phyla reported to possess copiotroph lifestyles [64, 91]. Synthetic fertilization positively correlates with *Proteobacteria* and *Actinobacteria* abundance [91–93]. Thus, we expected ASV269, ASV380 and ASV617 to be enriched at high fertilization. However, they were also enriched at low fertilization. Similar taxon shifts were reported by Fierer et al. [93] at the class level. While *Gammaproteobacteria* abundance increased at low and high nitrogen fertilization, *Alphaproteobacteria* abundance positively correlated with nitrogen fertilization rate. However, the observed response was dependent on soil source [93].

In contrast to the low fertilization, we found both depleted and enriched ASVs in the high fertilization treatment. Five ASVs were depleted at high fertilization (200 mg/L), including two *Proteobacteria* (ASV9 and ASV127) and three *Verrucomicrobiota* (ASV111, ASV191 and ASV493). ASV9 belong to the genus *Ramlibacter*, characterized by cyst-producing bacterial strains adapted to dessert environments whose cysts divide when water and nutrients are available [94, 95]. ASV127 belong to genus *Pseudolabrys,* and in contrast to our observations, its abundance in soil was positively correlated with increased fertilization [96, 97]*. Verrucomicrobia* community abundance is negatively correlated with low soil nutrient availability, and can serve as a bioindicator of soil fertility [98]. *Verrucomicrobia* has a slow-growing lifestyle adapted to thrive in poor-nutrient ecosystems [98]. Thus, when nutrients are optimum or in high concentration, *Verrucomicrobiae* can be outcompeted by bacteria with a fast growing habit like *Proteobacteria* and *Actinobacteria* [91]. Accordingly, we observed a depletion of three ASVs from the phylum *Verrucomicrobiae* in the high fertilization treatment *(*ASV111, ASV191 and ASV493*)*. ASV191 and ASV493 belong to the *Opitutaceae* family and were classified to the genus *Cephaloticoccus* and *Opitutus,* respectively. The genus *Opitutus* is commonly present in Sphagnum peat bogs and is involved in nitrate reduction and acetate and propionate production [65, 99, 100]. The genus *Cephaloticoccus* includes symbiotic bacteria living in ant guts [101]. Another example of bacteria from the *Opitutaceae* family living in insect guts is *Geminisphaera colitermitum* strain TAV2. TAV2 was isolated from termite guts (*Reticulitermes flavipe*) and potentially contributes to termite N-nutrition via dinitrogen fixation [102].

There were four ASVs enriched by the high fertilization treatment including three *Proteobacteria* (ASV14, ASV36, ASV60) and one *Verrucomicrobia* (ASV227). ASV14 belongs to the genus *Pseudaminobacter*. Species in *Pseudaminobacter* are sulfur-oxidant or pesticide degrading [103–106]. ASV60 belong to genus *Shinella*, whose members are known to fix nitrogen and degrade nicotine and chlorothalonil [107–109]. Both, *Pseudaminobacter* and *Shinella* species have been isolated from contaminated soil or sludge [106, 107]. ASV227 (family *Pedosphaeraceae*) did not follow the *Verrucomicrobia* oligotrophic lifestyle observed here and in other reports [98]. *Pedosphaeraceae* bacteria were reported to contribute to Cd remediation and plant growth promotion [110].

## Conclusion

In greenhouse container production of ornamental plants, root development is limited by the container size, and the primary source of microbial diversity is the substrate. This project provides an initial diversity profile of the bacterial communities occurring in a peat-based soilless substrate, and it contributes to our understanding of the effect of plant phenological stage and synthetic fertilizer rate on the soilless substrate microbiome in greenhouse production systems. High-throughput 16S rRNA amplicon sequencing identified in total 2,535 ASV in our peat-based substrate, but only 48 ASV belonging to 10 phyla were part of the core microbiome. Plant phenological stage and fertilization influenced the substrate microbiome composition and function. The most relevant chemical characteristics in our assessment of bacterial community composition were pH, TOC, and phosphate. Substrate pH and TOC were the most significantly associated with the observed community composition differences between phenological stages, and pH and phosphate helped to explain the differences across fertilizer levels. Development of microbial based solutions for the production of greenhouse ornamental plants requires a deep understanding of the microbial diversity existing in the soilless culture system and the factors modulating it and its functions. The relationship between substrate chemical properties and microbiome composition justifies future research to better understand their interactions and how they can be manipulated to promote the establishment of beneficial microbiomes or the efficacy of microbial based products.

### Supplementary Information

**Additional file 1. **Influence of sequencing depth on number of detected amplicon sequence variants (ASV) in substrate samples. Red and blue dashed lines represent sequencing depth of samples with lowest depth (L8 = 12,617 and L1 = 31,041). Samples were collected at budding (4 weeks after transplant) and full flowering (8 weeks after transplant), from container-grown *Impatiens walleriana* ‘Xtreme Red’ fertilized with a 15N-2.2P-12.5K-2.9Ca-1.2Mg water-soluble fertilizer at low (25 mg/L N), optimum (100 mg/L N), and high (200 mg/L N) levels.

**Additional file 2. **Fraction of reads identified by taxonomic level for each experimental condition. *Impatiens walleriana* ‘Xtreme Red’ were fertilized with 15N-2.2P-12.5K-2.9Ca-1.2Mg water-soluble fertilizer at 25, 100, or 200 mg/L of nitrogen (N). Samples were collected at budding and full flowering.

**Additional file 3: Table S1. **Nutritional content of substrate media extract.

## Data Availability

Amplicon sequencing data is available at the NCBI Sequence Read Archive (BioProject PRJNA1023591). The code to reproduce the microbiome data analysis can be found at https://github.com/DanielQuiroz97/Floral_Microbiome.

## Abbreviations

TOC: Total organic carbon
PGPR: Plant-growth promoting rhizobacteria.
SME: Saturated media extraction
EC: Electrical conductivity
TDN: Total dissolved nitrogen
ASV: Amplicon sequence variants
FDR: False discovery rate
CCA: Canonical correspondence analysis
AIC: Akaike Information Criterion
PCoA: Principal coordinate analysis
PERMANOVA: Multivariate analysis of variance
VIF: Variance inflation factor
F: Fluoride
Cl: Chloride
SO_4_: Sulfate
PO_4_: Phosphate
SynCom: Synthetic community

## References

[1] Carlile WR, Cattivello C, Zaccheo P (2015). Organic Growing Media: Constituents and Properties. Vadose Zo J.

[2] Marconi DJ, Nelson PV (1984). Leaching of applied phosphorus in container media. Sci Hortic (Amsterdam).

[3] Yeager TH, Barrett JE (1985). Phosphorus and sulfur leaching from an incubated superphosphateamended soilless container medium. HortScience.

[4] Henry JB, McCall I, Jackson B, Whipker BE (2017). Growth response of herbaceous ornamentals to phosphorus fertilization. HortScience.

[5] Siciliano A (2016). Assessment of fertilizer potential of the struvite produced from the treatment of methanogenic landfill leachate using low-cost reagents. Environ Sci Pollut Res.

[6] Fields JS, Owen JS, Altland JE (2021). Substrate stratification: Layering unique substrates within a container increases resource efficiency without impacting growth of shrub rose. Agronomy.

[7] Lin Y, Jones ML (2022). Evaluating the growth-promoting effects of microbial biostimulants on greenhouse floriculture crops. HortScience.

[8] South KA, Nordstedt NP, Jones ML (2021). Identification of plant growth promoting rhizobacteria that improve the performance of greenhouse-grown petunias under low fertility conditions. Plants.

[9] Pantigoso HA, Newberger D, Vivanco JM (2022). The rhizosphere microbiome: Plant–microbial interactions for resource acquisition. J Appl Microbiol.

[10] Bender SF, Schulz S, Martínez-Cuesta R, Laughlin RJ, Kublik S, Pfeiffer-Zakharova K (2023). Simplification of soil biota communities impairs nutrient recycling and enhances above- and belowground nitrogen losses. New Phytol..

[11] Panke-Buisse K, Poole AC, Goodrich JK, Ley RE, Kao-Kniffin J (2015). Selection on soil microbiomes reveals reproducible impacts on plant function. ISME J.

[12] Zaidi A, Khan MS, Ahmad E, Saif S, Rizvi A, Shahid M (2016). Growth stimulation and management of diseases of ornamental plants using phosphate solubilizing microorganisms: current perspective. Acta Physiol Plant.

[13] Berg G, Rybakova D, Fischer D, Cernava T, Vergès MCC, Charles T (2020). Microbiome definition re-visited: old concepts and new challenges. Microbiome.

[14] Banerjee S, van der Heijden MGA (2023). Soil microbiomes and one health. Nat Rev Microbiol.

[15] Philippot L, Raaijmakers JM, Lemanceau P, Van Der Putten WH (2013). Going back to the roots: The microbial ecology of the rhizosphere. Nat Rev Microbiol.

[16] Mengel K, Kirkby E (2001). Principles of Plant Nutrition.

[17] Canarini A, Kaiser C, Merchant A, Richter A, Wanek W (2019). Root exudation of primary metabolites: Mechanisms and their roles in plant responses to environmental stimuli. Front Plant Sci.

[18] Pascale A, Proietti S, Pantelides IS, Stringlis IA (2020). Modulation of the root microbiome by plant molecules: the basis for targeted disease suppression and plant growth promotion. Front Plant Sci.

[19] Van Deynze A, Zamora P, Delaux PM, Heitmann C, Jayaraman D, Rajasekar S (2018). Nitrogen fixation in a landrace of maize is supported by a mucilage-associated diazotrophic microbiota. PLoS Biol.

[20] De Zutter N, Ameye M, Bekaert B, Verwaeren J, De Gelder L, Audenaert K (2022). Uncovering new insights and misconceptions on the effectiveness of phosphate solubilizing rhizobacteria in plants: a meta-analysis. Front Plant Sci.

[21] Ali AM, Awad MYM, Hegab SA, El GAMA, Eissa MA (2021). Effect of potassium solubilizing bacteria (*Bacillus cereus*) on growth and yield of potato. J Plant Nutr.

[22] Radzki W, Gutierrez Mañero FJ, Algar E, Lucas García JA, García-Villaraco A, Ramos SB (2013). Bacterial siderophores efficiently provide iron to iron-starved tomato plants in hydroponics culture. Antonie Van Leeuwenhoek.

[23] Dai Z, Ahmed W, Yang J, Yao X, Zhang J, Wei L (2023). Seed coat treatment by plant-growth-promoting rhizobacteria Lysobacter antibioticus 13–6 enhances maize yield and changes rhizosphere bacterial communities. Biol Fertil Soils.

[24] Ahmed W, Dai Z, Zhang J, Li S, Ahmed A, Munir S (2022). Plant-microbe interaction: mining the impact of native bacillus amyloliquefaciens WS-10 on tobacco bacterial wilt disease and rhizosphere microbial communities. Microbiol Spectr.

[25] Pieterse CMJ, Zamioudis C, Berendsen RL, Weller DM, Van Wees SCM, Bakker PAHM (2014). Induced systemic resistance by beneficial microbes. Annu Rev Phytopathol.

[26] Roesch LFW, Fulthorpe RR, Riva A, Casella G, Hadwin AKM, Kent AD (2007). Pyrosequencing enumerates and contrasts soil microbial diversity. ISME J.

[27] Montagne V, Capiaux H, Barret M, Cannavo P, Charpentier S, Grosbellet C (2017). Bacterial and fungal communities vary with the type of organic substrate: implications for biocontrol of soilless crops. Environ Chem Lett.

[28] Valles-Ramirez S, Altland JE, Testen AL, Poelstra JW, Michel FC (2023). Microbial community structure in soilless substrates used for nursery crops. HortScience.

[29] Lopes LD, Hao J, Schachtman DP (2021). Alkaline soil pH affects bulk soil, rhizosphere and root endosphere microbiomes of plants growing in a Sandhills ecosystem. FEMS Microbiol Ecol.

[30] Fierer N, Jackson RB (2006). The diversity and biogeography of soil bacterial communities. Proc Natl Acad Sci U S A.

[31] Gibson JL, Nelson PV, Pitchay DS, Whipker BE (2001). Flower Growers’ Bulletin-February.

[32] Foster ZSL, Weiland JE, Scagel CF, Grünwald NJ (2020). The Composition of the fungal and oomycete microbiome of rhododendron roots under varying growth conditions, nurseries, and cultivars. Phytobiomes J.

[33] Cheng Z, Lei S, Li Y, Huang W, Ma R, Xiong J (2020). Revealing the variation and stability of bacterial communities in tomato rhizosphere microbiota. Microorganisms.

[34] Anzalone A, Mosca A, Dimaria G, Nicotra D, Tessitori M, Privitera GF (2022). Soil and soilless tomato cultivation promote different microbial communities that provide new models for future crop interventions. Int J Mol Sci.

[35] Edmonds JW, Sackett JD, Lomprey H, Hudson HL, Moser DP (2020). The aeroponic rhizosphere microbiome: community dynamics in early succession suggest strong selectional forces. Antonie van Leeuwenhoek.

[36] Bulgarelli D, Schlaeppi K, Spaepen S, Van Themaat EVL, Schulze-Lefert P (2013). Structure and functions of the bacterial microbiota of plants. Annu Rev Plant Biol.

[37] Vaccaro F, Cangioli L, Mengoni A, Fagorzi C (2022). Synthetic plant microbiota challenges in nonmodel species. Trends Microbiol.

[38] Warncke DD (1986). Analyzing greenhouse growth media by the saturation extraction method. HortScience.

[39] Landaverde AC, Shreckhise JH, Altland JE (2020). Storage procedures affect pH, electrical conductivity, and nutrient concentrations of pour-through leachate from pine bark and peat-based substrates. HortScience.

[40] Gohl DM, Vangay P, Garbe J, MacLean A, Hauge A, Becker A (2016). Systematic improvement of amplicon marker gene methods for increased accuracy in microbiome studies. Nat Biotechnol.

[41] Callahan BJ, McMurdie PJ, Rosen MJ, Han AW, Johnson AJA, Holmes SP (2016). DADA2: High-resolution sample inference from Illumina amplicon data. Nat Methods.

[42] Wright ES (2015). DECIPHER: Harnessing local sequence context to improve protein multiple sequence alignment. BMC Bioinformatics.

[43] Schliep KP (2011). phangorn: Phylogenetic analysis in R. Bioinformatics.

[44] Wang LG, Lam TTY, Xu S, Dai Z, Zhou L, Feng T (2020). Treeio: An R package for phylogenetic tree input and output with richly annotated and associated data. Mol Biol Evol.

[45] Yu G, Smith DK, Zhu H, Guan Y, Lam TTY (2017). Ggtree: an R package for visualization and annotation of phylogenetic trees with their covariates and other associated data. Methods Ecol Evol.

[46] Xu S, Dai Z, Guo P, Fu X, Liu S, Zhou L (2021). GgtreeExtra: compact visualization of richly annotated phylogenetic data. Mol Biol Evol.

[47] Kers JG, Saccenti E (2022). The power of microbiome studies: some considerations on which alpha and beta metrics to use and how to report results. Front Microbiol.

[48] Fedor P, Zvaríková M (2018). Biodiversity indices. Encyclopedia of ecology.

[49] Oksanen J, Blanchet FG, Kindt R, Legendre P, Minchin PR, O’hara R (2018). Package “Vegan”.

[50] Love MI, Huber W, Anders S (2014). Moderated estimation of fold change and dispersion for RNA-seq data with DESeq2. Genome Biol.

[51] Louca S, Parfrey LW, Doebeli M (2016). Decoupling function and taxonomy in the global ocean microbiome. Science.

[52] Neu AT, Allen EE, Roy K (2021). Defining and quantifying the core microbiome: Challenges and prospects. Proc Natl Acad Sci U S A.

[53] Xiong C, Singh BK, He JZ, Han YL, Li PP, Wan LH (2021). Plant developmental stage drives the differentiation in ecological role of the maize microbiome. Microbiome.

[54] Houlden A, Timms-Wilson TM, Day MJ, Bailey MJ (2008). Influence of plant developmental stage on microbial community structure and activity in the rhizosphere of three field crops. FEMS Microbiol Ecol.

[55] Alegria Terrazas R, Robertson-Albertyn S, Corral AM, Escudero-Martinez C, Kapadia R, Balbirnie-Cumming K (2022). Defining composition and function of the rhizosphere microbiota of barley genotypes exposed to growth-limiting nitrogen supplies. mSystems.

[56] Blok C, Eveleens B, van Winkel A (2021). Growing media for food and quality of life in the period 2020–2050. Acta Hortic.

[57] Fisher P (2007). Managing the pH of container media.

[58] Argo WR, Biernbaum JA (1996). The effect of lime, irrigation-water source, and water-soluble fertilizer on root-zone pH, electrical conductivity, and macronutrient management of container root media with impatiens. J Am Soc Hortic Sci.

[59] Rippy JFM, Nelson PV (2007). Cation exchange capacity and base saturation variation among Alberta, Canada, moss peats. HortScience.

[60] Fields JS, Fonteno WC, Jackson BE, Heitman JL, Owen JS (2014). Hydrophysical properties, moisture retention, and drainage profiles of wood and traditional components for greenhouse substrates. HortScience.

[61] Thompson LR, Sanders JG, McDonald D, Amir A, Ladau J, Locey KJ (2017). A communal catalogue reveals Earth’s multiscale microbial diversity. Nature.

[62] Hacquard S, Garrido-Oter R, González A, Spaepen S, Ackermann G, Lebeis S (2015). Microbiota and host nutrition across plant and animal kingdoms. Cell Host Microbe.

[63] Qu ZL, Sun H, Asiegbu FO, Kovalchuk A (2021). Microbiome of forest soil. Forest Microbiology: Volume 1: Tree Microbiome: Phyllosphere, Endosphere and Rhizosphere.

[64] Fierer N, Bradford MA, Jackson RB (2007). Toward an ecological classification of soil bacteria. Ecology.

[65] Dedysh SN, Pankratov TA, Belova SE, Kulichevskaya IS, Liesack W (2006). Phylogenetic analysis and in situ identification of Bacteria community composition in an acidic Sphagnum peat bog. Appl Environ Microbiol.

[66] Grunert O, Hernandez-Sanabria E, Vilchez-Vargas R, Jauregui R, Pieper DH, Perneel M (2016). Mineral and organic growing media have distinct community structure, stability and functionality in soilless culture systems. Sci Rep.

[67] Chaparro JM, Badri DV, Vivanco JM (2014). Rhizosphere microbiome assemblage is affected by plant development. ISME J.

[68] Yuan J, Zhao J, Wen T, Zhao M, Li R, Goossens P (2018). Root exudates drive the soil-borne legacy of aboveground pathogen infection. Microbiome.

[69] Dakora FD, Phillips DA (2002). Root exudates as mediators of mineral acquisition in low-nutrient environments. Plant Soil.

[70] Grunert O, Robles-Aguilar AA, Hernandez-Sanabria E, Schrey SD, Reheul D, Van Labeke MC (2019). Tomato plants rather than fertilizers drive microbial community structure in horticultural growing media. Sci Rep.

[71] Qiao Q, Wang F, Zhang J, Chen Y, Zhang C, Liu G (2017). The Variation in the Rhizosphere Microbiome of Cotton with Soil Type Genotype and Developmental Stage. Sci Rep.

[72] Leff JW, Jones SE, Prober SM, Barberán A, Borer ET, Firn JL (2015). Consistent responses of soil microbial communities to elevated nutrient inputs in grasslands across the globe. Proc Natl Acad Sci U S A.

[73] Tsai HH, Schmidt W (2017). Mobilization of iron by plant-borne coumarins. Trends Plant Sci.

[74] Pieterse CMJ, Stringlis IA (2023). Chemical symphony of coumarins and phenazines in rhizosphere iron solubilization. Proc Natl Acad Sci U S A.

[75] Finkel OM, Salas-González I, Castrillo G, Spaepen S, Law TF, Teixeira PJPL (2019). The effects of soil phosphorus content on plant microbiota are driven by the plant phosphate starvation response. PLoS Biol.

[76] Lundberg DS, Lebeis SL, Paredes SH, Yourstone S, Gehring J, Malfatti S (2012). Defining the core Arabidopsis thaliana root microbiome. Nature.

[77] Xu Y, Ge Y, Song J, Rensing C (2020). Assembly of root-associated microbial community of typical rice cultivars in different soil types. Biol Fertil Soils.

[78] Walters WA, Jin Z, Youngblut N, Wallace JG, Sutter J, Zhang W (2018). Large-scale replicated field study of maize rhizosphere identifies heritable microbes. Proc Natl Acad Sci.

[79] Stopnisek N, Shade A (2021). Persistent microbiome members in the common bean rhizosphere: an integrated analysis of space, time, and plant genotype. ISME J.

[80] Yee B, Oertli GE, Fuerst JA, Staley JT (2010). Reclassification of the polyphyletic genus Prosthecomicrobium to form two novel genera, Vasilyevaea gen. nov. and Bauldia gen. nov. with four new combinations: Vasilyevaea enhydra comb. nov., Vasilyevaea mishustinii comb. Int J Syst Evol Microbiol..

[81] Ravin NV, Rakitin AL, Ivanova AA, Beletsky AV, Kulichevskaya IS, Mardanov AV (2018). Genome analysis of *Fimbriiglobus ruber* SP5T, a planctomycete with confirmed chitinolytic capability. Appl Environ Microbiol.

[82] Kulichevskaya IS, Ivanova AA, Naumoff DG, Beletsky AV, Rijpstra WIC, Sinninghe Damsté JS (2020). Frigoriglobus tundricola gen. nov., sp. nov., a psychrotolerant cellulolytic planctomycete of the family Gemmataceae from a littoral tundra wetland. Syst Appl Microbiol..

[83] Rosenberg E, Rosenberg E, DeLong E, Lory S, Stackebrandt E, Thompson F (2014). The family Chitinophagaceae. The prokaryotes: other major lineages of bacteria and the archaea.

[84] Sangkhobol V, Skerman VBD (1981). Chitinophaga, a new genus of chitinolytic myxobacteria. Int J Syst Bacteriol.

[85] Wu X, Spencer S, Gushgari-Doyle S, Yee MO, Voriskova J, Li Y (2020). Culturing of “Unculturable” subsurface microbes: natural organic carbon source fuels the growth of diverse and distinct bacteria from groundwater. Front Microbiol.

[86] Liu QM, Ten LN, Im WT, Lee ST (2008). Castellaniella caeni sp. nov., a denitrifying bacterium isolated from sludge of a leachate treatment plant. Int J Syst Evol Microbiol..

[87] Spain AM, Peacock AD, Istok JD, Elshahed MS, Najar FZ, Roe BA (2007). Identification and isolation of a *Castellaniella* species important during biostimulation of an acidic nitrate- and uranium-contaminated aquifer. Appl Environ Microbiol.

[88] Friedrich MM, Lipski A (2008). Alkanibacter difficilis gen. nov., sp. nov. and Singularimonas variicoloris gen. nov., sp. nov., hexane-degrading bacteria isolated from a hexane-treated biofilter. Int J Syst Evol Microbiol.

[89] Peng M, Jia H, Wang Q (2017). The effect of land use on bacterial communities in saline-alkali soil. Curr Microbiol.

[90] Chen RW, He YQ, Cui LQ, Li C, Shi SB, Long LJ (2021). Diversity and distribution of uncultured and cultured gaiellales and rubrobacterales in South China Sea sediments. Front Microbiol.

[91] Dai Z, Su W, Chen H, Barberán A, Zhao H, Yu M (2018). Long-term nitrogen fertilization decreases bacterial diversity and favors the growth of Actinobacteria and Proteobacteria in agro-ecosystems across the globe. Glob Chang Biol.

[92] Pan Y, Cassman N, de Hollander M, Mendes LW, Korevaar H, Geerts RHEM (2014). Impact of long-term N, P, K, and NPK fertilization on the composition and potential functions of the bacterial community in grassland soil. FEMS Microbiol Ecol.

[93] Fierer N, Lauber CL, Ramirez KS, Zaneveld J, Bradford MA, Knight R (2012). Comparative metagenomic, phylogenetic and physiological analyses of soil microbial communities across nitrogen gradients. ISME J.

[94] Heulin T, Barakat M, Christen R, Lesourd M, Sutra L, De Luca G (2003). Ramlibacter tataouinensis gen. nov., sp. nov., and Ramlibacter henchirensis sp. nov., cyst-producing bacteria isolated from subdesert soil in Tunisia. Int J Syst Evol Microbiol..

[95] De Luca G, Fochesato S, Lavergne J, Forest KT, Barakat M, Ortet P (2019). Light on the cell cycle of the non-photosynthetic bacterium *Ramlibacter tataouinensis*. Sci Rep.

[96] Villamil MB, Kim N, Riggins CW, Zabaloy MC, Allegrini M, Rodríguez-Zas SL (2021). Microbial signatures in fertile soils under long-term N management. Front Soil Sci.

[97] Chaudhari D, Rangappa K, Das A, Layek J, Basavaraju S, Shouche Y, et al. Homogenization of Rhizosphere Bacterial Communities by Pea (*Pisum sativum* L.) 2 Cultivated under Different Conservation Agricultural Practices in the Eastern Himalayas. bioRxiv. 2019;1-39.

[98] Navarrete AA, Soares T, Rossetto R, van Veen JA, Tsai SM, Kuramae EE (2015). Verrucomicrobial community structure and abundance as indicators for changes in chemical factors linked to soil fertility. Antonie van Leeuwenhoek.

[99] Chin KJ, Janssen PH (2002). Propionate formation by *Opitutus terrae* in pure culture and in mixed culture with a hydrogenotrophic methanogen and implications for carbon fluxes in anoxic rice paddy soil. Appl Environ Microbiol.

[100] Chin KJ, Liesack W, Janssen PH (2001). Opitutus terrae gen. nov., sp. nov., to accommodate novel strains of the division “Verrucomicrobia” isolated from rice paddy soil. Int J Syst Evol Microbiol.

[101] Lin JY, Russell JA, Sanders JG, Wertz JT (2016). Cephaloticoccus gen. Nov., a new genus of ‘Verrucomicrobia’ containing two novel species isolated from Cephalotes ant guts. Int J Syst Evol Microbiol.

[102] Wertz JT, Kim E, Breznak JA, Schmidt TM, Rodrigues JLM (2012). Genomic and physiological characterization of the Verrucomicrobia isolate Diplosphaera colitermitum gen. nov., sp. nov., reveals microaerophily and nitrogen fixation genes. Appl Environ Microbiol.

[103] Alam M, Roy C, Pyne P, Agarwal A, George A, Ghosh W (2012). Whole-genome shotgun sequence of the sulfur-oxidizing chemoautotroph *Pseudaminobacter salicylatoxidans* KCT001. J Bacteriol.

[104] Zhang R, Cui Z, Jiang J, He J, Gu X, Li S (2005). Diversity of organophosphorus pesticide-degrading bacteria in a polluted soil and conservation of their organophosphorus hydrolase genes. Can J Microbiol.

[105] Kim H, Kim DU, Lee H, Yun J, Ka JO (2017). Syntrophic biodegradation of propoxur by Pseudaminobacter sp. SP1a and Nocardioides sp. SP1b isolated from agricultural soil. Int Biodeterior Biodegrad.

[106] Xia X, Li J, Zhou Z, Wang D, Huang J, Wang G (2018). High-quality-draft genome sequence of the multiple heavy metal resistant bacterium *Pseudaminobacter manganicus* JH-7. Stand Genomic Sci.

[107] Mesnage R, Teixeira M, Mandrioli D, Falcioni L, Ducarmon QR, Zwittink RD, et al. Shotgun metagenomics and metabolomics reveal glyphosate alters the gut microbiome of Sprague-Dawley rats by inhibiting the shikimate pathway. bioRxiv. 2019;1-33.

[108] Taulé C, Mareque C, Barlocco C, Hackembruch F, Reis VM, Sicardi M (2012). The contribution of nitrogen fixation to sugarcane (Saccharum officinarum L.), and the identification and characterization of part of the associated diazotrophic bacterial community. Plant Soil.

[109] Taulé C, Castillo A, Villar S, Olivares F, Battistoni F (2016). Endophytic colonization of sugarcane (Saccharum officinarum) by the novel diazotrophs Shinella sp. UYSO24 and Enterobacter sp. UYSO10. Plant Soil.

[110] Yuan Q, Wang P, Wang X, Hu B, Tao L (2022). Phytoremediation of cadmium-contaminated sediment using *Hydrilla verticillata* and *Elodea canadensis* harbor two same keystone rhizobacteria *Pedosphaeraceae* and *Parasegetibacter*. Chemosphere.

